# Irritable Bowel Syndrome and Resilience

**DOI:** 10.3390/jcm12134220

**Published:** 2023-06-22

**Authors:** Mihaela Fadgyas Stanculete, Abdulrahman Ismaiel, Stefan-Lucian Popa, Octavia Oana Capatina

**Affiliations:** 1 Department of Neurosciences, “Iuliu Hatieganu” University of Medicine and Pharmacy, 400006 Cluj-Napoca, Romania; 2 2nd Department of Internal Medicine, “Iuliu Hatieganu” University of Medicine and Pharmacy, 400006 Cluj-Napoca, Romania

**Keywords:** irritable bowel syndrome, resilience, stress, disorders of the brain–gut interaction, quality of life

## Abstract

Irritable bowel syndrome (IBS) is a disorder of the gut–brain interaction (DGBI), characterized, mainly in severe cases, by altered psychological stress reactivity, psychological disorders, and dysfunction of the brain–gut–microbiota axis. Prior studies have highlighted significant physical and emotional impairments in the health-related quality of life of patients with IBS. Resilience is a psychosocial ability that reduces negative emotions while enhancing adaptation to adversities. Resilience is essential for health promotion and stress response. The present study aimed to carry out a review of the literature in multiple databases, using the descriptors “resilience”, “resiliency”, and “irritable bowel syndrome”. The inclusion criteria for obtaining the most relevant papers were research articles on resilience and irritable bowel syndrome written in English, published in a peer-reviewed journal, and involving human subjects. Studies specifically on resilience in IBS were sparse. These results need to be understood in light of these limitations. As resilience appears to be modifiable, it is essential to conduct direct research on resilience-enhancing interventions for people with IBS. The study of the factors involved in successful adaptation must be extended, to possibly yield new interventions that help the patients overcome the difficulties imposed by the disease.

## 1. Introduction

Resilience is a broad term that subsumes several conceptually related notions. It comprises factors such as the tolerance of negative effects, positive acceptance of change, perceived control, and personal competence [1,2,3]. Resilience, defined as the ability to recover from or adjust easily to a stressful event, has been related to social support that works as an external coping mechanism, favoring the activation of internal coping and the capacity of the organism to react. Measurement of stress-resilience levels is essential in screening the general population for individuals at risk of developing stress-induced somatic and psychological disorders. 

Modern resilience research started in the ‘80s and continued in the ‘90s with studies of psychopathological outcomes among children who experienced early life adversities, analysis concerning the adjustment to life-threatening adult disorders, and the reaction to acute traumatic events [4,5,6]. Researchers have been interested in explaining how individual, family, and societal characteristics contribute to mental health [7,8]. Furthermore, individual strengths and capacities are recognized as health-promoting assets [9]. 

The well-known axiom of stress research states that distress arises when the demands of a situation exceed the individual’s coping resources [10]. Resilience is present if appraisal processes signal that the demands of the problematic situation are balanced with accessible coping resources. Therefore, resilience represents a focus of attention of research related to stress. 

However, in recent years, research interest have shifted to neurobiological and psychological factors and mechanisms that characterize resilient individuals [11,12,13]. These studies have highlighted many ways in which individuals understand and build resources. 

Resilient people are more flexible and able to cope using several protective resources within themselves or their environment. Achieving resilient functioning depends on different factors. The most important seems to be one’s ability to recruit resilience resources within oneself and from positive social connections within and between social networks that facilitate adaptive coping responses [14,15,16,17,18,19]. In response to adversity, patients mobilize physiological, affective, cognitive, and social resources in response to the distress.

In clinical research, it is essential to have exact and clear conceptual definitions of resilience’s theoretical and practical aspects. Research has demonstrated a connection between the indicators of positive emotions and resilience in various contexts, including chronic pain. Positive affect is generally considered a state variable that can bolster resilience to a forthcoming stressor, and resilience is measured in terms of the capacity to respond to a stressor. Resilience, as a trait measure, should be able to predict emotional and physiological responses to stressors that are distinct from current emotional states. In this context, an individual’s resilience can be gauged by examining their physical or mental condition after exposure to certain stressors. However, it can also be evaluated more generally as a trait based on their self-reported reactions to typical stressors. Moreover, using measures that assess these resilience-related conductive qualities provides an opportunity to gain insight into broader facets of resilience that are not revealed by the traditional model, which concentrates on the level of psychopathology after adversity.

One definition of resilience refers to outcomes resulting from stressful situations. Other definitions refer to resilience as a particular type of response to stressors, or resilience could be considered a combination of protective factors [20]. A new line of approach to resilience is the conceptualization of resilience as involving three components: recovery (the return to baseline functioning following a significant stressor), sustainability (the ability to maintain functionality during periods of stress), and growth (heightened adaptation beyond previous levels of functioning) [21,22]. 

Psychosocial adjustment to IBS symptoms can be integrated into an approach that describes the complex nature of an individual’s emotional and behavioral responses to illness. Clinical experience and research suggest that patients’ experiences of IBS symptoms are highly varied [23,24]. This variability may be due to the influence of individual psychological and social factors. Studies exploring the subjective factors that shape an individual’s reaction to IBS have typically concentrated on attachment styles, personality types, coping styles, optimism/pessimism, and resilience styles [25,26,27]. Numerous theories on what contributes to the emergence of resilience have been investigated and researchers have yet to arrive at a definite agreement. 

Coping, a construct related to resilience, is represented by the use of different cognitive and behavioral strategies aimed at reducing distress, which is often associated with negative experiences, such as chronic illnesses. Furthermore, resilience can be conceptualized as a measure of stress-coping ability in response to adversity [28]. 

Coping styles represent cognitive and behavioral changes resulting from managing an individual’s specific external or internal stressors. These can be classified into two categories: active and passive.

Active coping responses are intentional efforts of the subject to minimize a stressor’s physical, psychological, or social harm and are associated with actual or perceived control over the stressor. Coping mechanisms, such as active participation or engagement in one’s care, lead to changes that facilitate an adaptive, resilient response [29,30]. 

In contrast, passive coping includes mechanisms such as avoidance or helplessness, and is associated with increased vulnerability [31]. 

Although some coping strategies may be considered more effective, their efficacy varies depending on the case. In conclusion, coping is not a one-time effort that a person takes, but rather a set of reactions taking place over time by which the stressors and the individual influence each other. Various studies have pointed out that particular coping strategies are relevant to resilience [32].

Irritable bowel syndrome is a multifactorial, chronic relapsing disorder characterized by gastrointestinal symptoms that substantially impair patients’ quality of life, affecting or associated with psychological, physical, and social functions. IBS’s symptom variety and chronic pain fit into a biopsychosocial model [33,34,35].

Enhanced stress responsiveness is a potential mechanism involved in the pathophysiology of IBS. Both the hypothalamic-pituitary-adrenal (HPA) axis and sympathetic nervous system can modulate mucosal immune function. Enhanced stress responsiveness represents one possible mechanism involved in the pathophysiology of IBS. Previous research has shown that alterations in adrenocorticotropin hormone (ACTH), cortisol, and catecholamine levels are present in IBS and several other stress-sensitive disorders, including anxiety and depression. Increased mucosal immune activation and plasma cytokine levels could affect HPA axis stimulation, resulting in a hyper-responsive HPA axis [36,37].

Resource availability and coping capacity are factors that modify outcomes, particularly mental health, in the presence of IBS symptoms. Currently, those neurobiological factors that lead to increased vulnerability to stress in some IBS patients and increased resilience in others are still an area of active research.

IBS symptoms adversely influence the range of life domains of people living with the illness. For example, psychological distress is frequently observed in irritable bowel syndrome, impacting functioning, quality of life (QOL), illness cost, and indirectly, the economy (through absenteeism and reduced productivity in the workplace) [38,39,40,41]. The financial burden resulting from functional impairments and possible social consequences of IBS as a chronic disease has justified the interest in evaluating coping mechanisms and, in the recent past, the study of resilience resources.

Over the past decade, many studies have shown that resilience predicts mental health indicators (life satisfaction, anxiety, depression, positive affect) in chronic illnesses [42,43,44,45]. IBS patients with psychiatric comorbidities, such as depression or anxiety, are at a higher risk of costing the health system than other patients because of the expense of unplanned emergency visits and hospital admissions. Many researchers have examined various psychological aspects and reported associations with or differences in emotional distress and coping [46,47,48,49,50]. Psychological management strategies for IBS have not yet been established as precise intervention methods, and attention to the psychological problems of patients with IBS and related social issues remains limited [51,52].

However, designing appropriate interventions to improve the psychological condition of patients with IBS requires preliminary analyses of the protective factors of this disease. Therefore, we conducted a literature review on IBS and resilience to determine what factors have been reported to predict, promote, or associate with resilience, and the impacts of different treatments on resilience. Moreover, we tried to answer two questions: how resilience can be measured in individuals at risk of developing stress-induced somatic and psychological disorders, and what are the specific resilient-enhancing interventions for people with IBS?

## 2. Materials and Methods

The objective of this review was to explore the evidence for resilience in IBS patients and evaluate the effectiveness of interventions designed to increase resilience in individuals with IBS reported in the literature.

### Study Selection and Data Extraction

Studies were identified using multiple online databases and manual searching. We searched for articles indexed in the PubMed, Scopus, EMBASE, EBSCOhost, and Web of Science databases. The search terms included (resilience OR resilience strategies OR resiliency) AND (irritable bowel syndrome OR IBS). We did not include “coping” in the search terms because recent conceptualization has shown resilience to be a multifactorial construct that consists of a mixture of individual internal attributes, including self-efficacy, determination, temperament, aptitude, the availability or access to external resources such as social support and consequences such as effective coping behavior [53].

Review articles, dissertations, case reports, letters to the editor, editorials, articles not written in English, and case studies were excluded. All articles were selected from peer-reviewed journals. Titles and abstracts were screened for eligibility by two independent authors (M.F.S. and O.C.) followed by an evaluation of full texts of the articles that fulfilled the inclusion and exclusion criteria. Data extraction was performed independently by both reviewers. Finally, references of the included papers were manually searched for additional articles.

After eligible studies were assessed and data extraction performed, all the discrepancies in extracted data were resolved through discussion to reach a consensus. Extracted data on authors’ names, year of publication, country or study population, sample size, study design, and the resilience strategy used in IBS were reported in Table 1. A systematic review or meta-analysis of the results was not feasible because of the clinical and statistical heterogeneity of the existing studies [54,55,56,57,58,59,60,61,62,63,64,65].

## 3. Results

A total of 256 articles were identified and retrieved. All papers were selected from peer-reviewed journals. After removing duplicates, 249 articles were screened, 192 were excluded by title and abstract, and 57 were examined in full. Another 46 papers were removed for different reasons: unrelated to the subject, only abstracts, letters to the editor, animal studies, and languages other than English. Thus, 11 articles (10 quantitative and 1 qualitative method) were included in this review. The authors, sample size, study design, statistical method used, the aim of the study, and summary of results of the included articles are listed in Table 1.

As articles varied significantly in terms of methodology, both qualitative and quantitative research designs included measures of resilience, aims, and purposes; a narrative synthesis of the literature to gather information from various approaches is described in this article. Furthermore, narrative synthesis is helpful when other methods are unsuitable because of the wide range of methodologies of the studies being reviewed.

### 3.1. Design of Studies

The sample sizes of the populations relevant for the present review, that is, IBS patients, ranged from 19 to 820 with a mean sample size of 142, highlighting the notable differences between studies. Table 1 summarizes the methodologies of the included studies, of which three were interventional studies, seven were observational studies, nine were cross-sectional, and one was prospective longitudinal.

The resilience measure used in six studies was the Connor–Davidson Scale (CD-RISC), with its 10-item and 25-item versions. Another study used an 8-item German version of the Resilience Scale, and a 25-item Resilience Coping Scale (RSC) was found in another study [54]. In addition, Ringstorm et al. used a semi-structured interview design to allow participants to share their personal experiences with a diagnostic workup [55]. Furthermore, the remaining two studies provided no direct measure of resilience; one used the Lang and Goulet Hardiness Scale (LGHS) and the other the Pain Catastrophizing Scale (PCS) to assess resilience’s actual ability to adapt to symptoms of the disease [56,57,58,59].

### 3.2. Outcome Measures

#### 3.2.1. Comparison of Resilience Scores

Three studies compared psychological resilience between IBS patients and the general population or healthy matched controls. All three studies showed significant differences in the mean resilience scores, with IBS patients having lower scores than healthy controls. Park et al. reported that after adjusting for sex, age, and education, IBS patients had significantly lower mean resilience scores compared to healthy controls, as measured by the CD-RISC (72.16 ± 14.97 vs. 77.32 ± 12.73, *p* = 0.003) and BRS (3.29 ± 0.87 vs. 3.93 ± 0.69, *p* < 0.001) [60]. Shahdadi, et al., reported a significant difference in the mean resilience score with lower scores in IBS patients [61]. In a study conducted by Parker, et al., respondents with IBS had significantly lower mean resilience scores than the general population after controlling for age, sex, income, employment status, education, military status, marital status, ethnicity, and race, as measured by the CD-RISC [64].

#### 3.2.2. Factors Associated with Resilience

A study by Palgi, et al. aimed to explore the psychosocial factors associated with psychological distress in IBS patients and the contribution of cognitive appraisal to adjustment. This study showed that psychological distress and depressive symptoms among IBS patients are better-predicted by their global positive illness cognition appraisal, specific illness cognition appraisal of helplessness, resilience, and, to a lesser extent, by social support, perceived optimism, illness cognitions, appraisals of acceptance, and perceived benefit. In addition, the results of multiple mediation tests of indirect effects showed that resilience was a significant mediator when depressive symptoms were the dependent variable. Furthermore, the results of the multiple mediation tests of indirect effects, when psychological distress was the dependent variable, showed that resilience was again a significant mediator [58].

Another study that aimed to assess the effect of selected personality traits and stress on IBS symptoms showed a correlation between resilience and emotion-oriented coping, and that patients with IBS had a lower level of perceived self-efficacy and resilience [63].

Fisher, et al. proposed a multi-dimensional stress model, which posits that childhood trauma increases adult stress reactivity and reduces resilience. Following the proposed conceptual model, this study showed that childhood trauma positively affected stress reactivity and negatively impacted resilience, which predicted the manifestation of functional somatic syndrome, including IBS, via chronic stress. In addition, an indirect effect of childhood trauma was observed in the development and perpetuation of functional somatic syndrome via chronic stress. However, there is no direct link between traumatic experiences and functional somatic syndromes [55].

A cross-sectional study that included 123 subjects investigated the role of positive personality traits (psychological hardiness and interpersonal forgiveness) in the emotional regulation of patients with IBS. Regression analyses performed showed that personality traits, hardiness and forgiveness acted as protective factors for emotional dysregulation [57].

Another study that adopted a qualitative methodology called interpretative phenomenological analysis included a sample of 20 patients who completed a semi-structured interview. The semi-structured interview was designed to capture participants’ lived experiences and allow them to share their experiences. From the analysis of interviews conducted through interpretative phenomenological analysis, one master theme emerged: validation of IBS experience, which was inferred from three subthemes: the duality of suffering in IBS, coping with inflicted discomfort and pain, and capacity for resilience [54].

#### 3.2.3. Interventions

All interventions aimed at improving resilience in IBS patients were psychological.

A quasi-experimental study, which included two intervention and control groups, conducted by Haghayegh et al., aimed to determine the efficacy of dialectical behavioral therapy (DBT) on resilience in IBS and showed no significant difference between groups. However, despite no significant difference between the groups, the increase in resilience in the DBT group was greater than in the control group [59].

Another study aimed to investigate changes in resilience in IBS patients after gut-directed hypnotherapy (GDH). In this trial, 52 patients were enrolled and assigned to two experimental and control groups [62]. The results showed that greater resilience, better quality of life, lower symptom severity, and psychological distress were observed after GDH intervention.

Kuo and colleagues evaluated the impact of mind–body interventions (MBIs) on 19 IBS patients enrolled in a nine-week relaxation response-based mind–body group intervention (RR-MBI). Resilience was not a direct measure of the study, but the adjustment was assessed using the Pain Catastrophizing Scale scores, which improved significantly post-intervention at short-term follow-up [56].

## 4. Discussion

Very few published studies on resilience and IBS were found in our literature review. A lack of randomized clinical trials that have studied resilience in IBS was observed because this topic is still new, albeit increasingly attractive to researchers. Additionally, the articles included in the analysis were predominantly descriptive methodological, and cross-sectional.

Patients with IBS showed significantly lower resilience than the general population did. Moreover, lower resilience was associated with worse IBS symptom severity.

IBS frequent attenders (FA) are a particular population that may represent a problem for general practitioners (GPs), consuming much of the resources and increasing their workload.

Furthermore, FAs appear to be characterized by a low sense of coherence, low internal locus of control, and increased risk of disability. Frequent attendance in primary care was associated with impairments in QoL and high clinical complexity characterized by physical and psychiatric comorbidities [66].

While general practitioners (GPs) play a crucial role in managing IBS owing to the condition’s high prevalence, there can be certain limitations compared to specialized gastroenterologists. Gastroenterologists may have more extensive knowledge and experience in diagnosing IBS and are likely to be more familiar with the Rome IV criteria, which are widely used for IBS diagnosis and have the advantage of being able to refer patients for specialized investigations, such as a colonoscopy. With their specialized training, they may have a deeper understanding of other gastrointestinal conditions that can mimic IBS symptoms, such as inflammatory bowel disease (IBD), celiac disease, or gastrointestinal malignancies. Furthermore, they can collaborate with other specialists, such as dietitians or psychologists, who may provide additional support in managing IBS.

On the other hand, GPs may have limited access to these resources, which can impact the overall management of the condition. Although GPs are often the first point of contact for patients with IBS, they may have a different level of expertise and follow-up than specialized gastroenterologists.

A study by Belinni et al. [67] aimed to evaluate the clinical features of IBS patients and the approach of GPs in Italy for managing IBS. The study involved 28 GPs who completed a questionnaire, and the analysis included 229 patients. The results indicated that only 35.7% of GPs were familiar with the Rome II criteria commonly used for diagnosing IBS. The most prevalent symptoms reported by the patients were changes in bowel habits and abdominal pain/discomfort. Constipation was more frequently observed as the primary symptom (74.2%) than diarrhea. Routine blood tests (76.4%) and abdominal ultrasounds (42.2%) were requested more often than colonoscopies (31.1%). Specialist consultations were recommended for 63.3% of the patients. In terms of treatment, antispasmodics were the most frequently prescribed drugs, especially for diarrhea (91.4%), while constipation was treated with antispasmodics less frequently (55.7%). The study concluded that GPs needed more familiarity with the Rome II criteria for diagnosing IBS [67].

Diagnostic tests and specialist consultations are commonly recommended, and antispasmodics are frequently prescribed. The researchers suggested the collaborative development of guidelines by GPs and gastroenterologists to manage patients with IBS at a lower cost-effectively.

The study highlighted the need for improved familiarity with diagnostic criteria among GPs. It emphasized the importance of joint guideline development by GPs and specialists to optimize the management of IBS patients by GPs and specialists [67].

It is essential to note the variations in the knowledge and expertise of individual doctors, including GPs, who have a particular interest in gastroenterology. Collaborative efforts between GPs and gastroenterologists can also enhance the quality of care provided to IBS patients. Ultimately, the choice of healthcare provider depends on the severity and complexity of the condition and the individual patient’s preferences and access to specialized care.

Several studies have identified a high degree of unmet health needs for FAs, underlining the importance of understanding the patient with their context, not only focusing on symptoms. Therefore, evaluating undetected and/or mistreated psychiatric comorbidities could improve the global functioning of these patients [58,59].

A number of studies have proposed a conceptual model, supported by empirical evidence, which can be used to guide patient education/intervention and clinical trials to determine the degree to which developing resilience can improve affect and, consequently, reduce the severity of IBS symptoms [60,61,62,63,64,65,66].

As expected, psychological distress and depressive symptoms are associated with resilience, supporting the evidence that personal resources help individuals cope with stressors and account for individual adjustment to illness. Moreover, these findings are consistent with previous studies showing that subjects without resilient adaptation present difficulties regulating negative emotions (anger, depression, anxiety) and increased sensitivity to adverse life events [60,61,62,63].

The factors identified in this review as interfering with resilience have previously been addressed through psychosocial interventions, including cognitive behavioral therapy, dialectical behavior therapy, gut-directed hypnotherapy, the use of central neuromodulators for anxiety/depression management, stress management, and psychoeducation [64,65,66,67]. The psychosocial variables that have already been investigated in cross-sectional studies are worth exploring with longitudinal designs in future research.

Furthermore, the consequences of depression and anxiety on IBS-related outcomes have recently received increasing attention. For example, psychological distress symptoms may be independently associated with health-related quality of life in patients with IBS but also with lower resilience, leading to a lower quality of life [64].

The treatment of IBS with central and peripheral neuromodulators will be more successful when tailored to individual patient requirements. Although no studies have evaluated the effect of medication on resilience, neuromodulators are essential medications if proper indications are endorsed, especially when the patient is experiencing more intense pain, acting on multiple levels.

Previous research has shown that IBS is a contributing factor to sleep disorders [68]. People with IBS often experience difficulty sleeping, frequent waking, and difficulty falling back asleep after waking. Studies using both self-reported and objective metrics have demonstrated that inadequate sleep quality is a significant predictor of the following day’s IBS symptoms (e.g., abdominal pain/discomfort), psychological distress (e.g., depression and anxiety), and daytime impairment (e.g., fatigue and sleepiness) [69]. Although no studies have looked explicitly at IBS patients, other studies have proposed that depressive symptoms mediate the link between emotion-focused and problem-focused coping and sleep issues [70].

In addition to IBS, other disorders of the gut–brain interaction (DGBI) have been associated with the concepts of coping and resilience, mainly because they are inextricably linked to their psychological impact [71]. Resilience is linked to the normal functioning of the central nervous system and the gut–brain axis, and neurotransmitters and neuromodulators such as dopamine, serotonin, corticotropin-releasing factor, cortisol, and noradrenaline have been demonstrated to be involved in the connection between resilience and acute stress [72,73,74,75,76,77,78,79,80,81]. It has been demonstrated by Park et al. that patients with functional gastrointestinal diseases have elevated corticoids and reduced resilience when compared to healthy individuals [60].

Stress-resilience is affected by a variety of psychological and biological elements, including the microbiome–gut–brain axis [73]. Additionally, Marin et al. conducted several studies to explore the connection between alterations in gut microbiota caused by stress and resilience. They observed distinctions between those who are stress-resilient and those who are stress-sensitive [74].

Some limitations of this review must be mentioned. Most studies included a selected population of IBS patients visiting a gastroenterology department and were not necessarily representative of the general population. Specifically, IBS individuals have different psychological characteristics from IBS sufferers who do not attend tertiary gastroenterology clinics. Potentially, help-seeking IBS patients differ in personality traits and coping mechanisms from non-help-seeking IBS patients. Consequently, due to the selected sample of IBS patients used in the studies included in our review, the generalization of outcomes is hampered. Another limitation of this study is represented by the fact that only one qualitative study was included because these could not be found, and there was an absence of a more significant number of randomized and controlled clinical trials.

Potential sources of bias in the surveys about resilience include a non-representative sample that cannot be applied to the broader population (e.g., frequent attenders), gender bias (most participants are females), missing data in the items, and recall bias (the tendency to overestimate or underestimate past positive or negative emotional experiences).

Given the lack of efficient medication capable of controlling IBS symptoms, there is a need to discover new therapeutic targets and strategies to improve adaptation to illness symptoms. Unfortunately, the resilience of IBS patients has been poorly studied by healthcare professionals. A poor concept definition and lack of a unified methodology impede up-to-date resilience research. Further efforts to incorporate psychological and neurobiological mechanisms implicated in resilience are required to surmount the fragmentation of this construct, leading to the discovery of new interventions to promote mental health and well-being.

Moreover, it is necessary to develop new research to understand health professionals’ perspectives on the importance of resilience in the assistance provided to IBS patients, which will favor the clinical application of this phenomenon. These limitations can be overcome by projecting larger RCTs with larger samples of IBS patients. They must explicitly clarify that resilience represents competent adaptation in the face of adversity, where resilience should also be defined as a phenomenon and not just a personal trait. Prospective and longitudinal designs are strongly recommended for studying resilience in this category of patients.

Addressing relevant psychosocial factors in IBS patients and their life contexts is essential because it may provide a clear rationale for implementing effective interventions aimed at increasing adjustment to illness. The involvement of an interdisciplinary team can successfully reduce distress and maximize resilience.

## 5. Conclusions

Resilience is a multifaceted response to a patient’s role. Various interrelated factors influence individual responses and psychosocial factors are of prime importance. Unfortunately, up to this point, evidence for resilience-enhancing interventions on gastrointestinal symptoms and psychological health status remains unsatisfactory. Considering the growing interest in resilience and the suggested benefits to patients in illness self-management and their overall health, the absence of published studies indicates that further research is required to broaden our understanding. There is a need to untangle psychosocial constructs concerning IBS resilience using validated resilience measures and large sample sizes, bearing in mind that factors that promote and protect resilience unfold over the life course.

## Figures and Tables

**Table 1:** This table presents the summary of the authors, sample size, study design, statistical method used, the aim of the study, and summary of results of the included articles.

First Author/Year	Sample (*n*)	Methodology	Resilience Measure	Method of Data Analysis	Resilience Findings	Main Study Aim
Ringstrom, G., 2013 [54]	IBS *n* = 20	Interpretative phenomenological analysis.	Semi-structured interviews that provided participants with an opportunity to share their personal experiences of the diagnostic workup.	Interviews were read systematically to identify themes.	Validation of IBS experience comprising three superordinate themes: - The duality of suffering in IBS; - Coping with inflicted discomfort and pain; - Increased capacity for resilience.	To explore experiences of undergoing a diagnostic workup in patients with IBS.
Fischer, S., 2014 [55]	*n* = 39	Web survey, cross-sectional and longitudinal	8 items from the German version of the Resilience Scale	Structural equation modeling	Occurrence of childhood trauma is significantly related to elevated stress reactivity and attenuated resilience, which in turn predicted the manifestation of FSS via chronic stress.	Proposal of a multi-dimensional stress model which posits that childhood trauma increases adult stress reactivity and reduces resilience.
Kuo, B., 2015 [56]	IBS *n* = 19 IBD *n* = 29	Interventional study	No direct resilience measure. PCS evaluated adjustment	One-way ANOVA, Fischer exact test, mixed linear models	PCS score improved significantly post-intervention at short-term follow up for IBS patients.	The impact of a body-and-mind intervention on IBS.
Mazaheri, M., 2015 [57]	IBS *n* = 123	Cross-sectional study	No direct measure of resilience. LGHS assessed adjustment.	Descriptive analysis, Pearson correlation coefficient, Multivariate and Binary Logistic regression analyses	Traits of hardiness were considered protective factors for emotional dysregulation.	To investigate the role of positive personality traits (psychological hardiness and interpersonal forgiveness) in emotion regulation in IBS.
Palgi, S., 2015 [58]	IBS *n* = 103	Cross-sectional study	CD-RISC	Hierarchical multiple regression	Resilience is one of the prediction factors for psychological distress and depressive symptoms among IBS patients.	To explore the psycho-social factors that are associated with psychological distress among IBS patients and to gain more information on psychiatric symptoms and cognitive attitudes.
Abbas Haghayegh, S.,2017 [59]	IBS *n* = 52	Quasi-experimental	25-item CD-RISC	Descriptive statistics. MANOVA	Dialectical behavioral therapy: no significant effect on resilience in IBS patients.	To determine the efficacy of dialectical behavioral therapy on stress, resilience and coping strategies of IBS patients.
Park, S.H., 2017 [60]	IBS *n* = 154 HC *n* = 102	Cross-sectional study	CD-RISC BRS	Descriptive statistics, Wilcoxon or Chi-square tests, regression analysis	Resilience was significantly lower in IBS compared to HCs.	To compare resilience in IBS and HCs to assess its relationships with IBS symptom severity, quality of life (QOL), EALs, and HPA axis response.
Shahdadi, H., 2017 [61]	IBS *n* = 50 Matched controls *n* = 50	Analytical comparative study	25-item CD-RISC	Descriptive statistics, MANOVA	Difference between resilience and the components of positive relations with others, environmental mastery, purpose in life and acceptance in IBS women and healthy women.	To compare resilience and psychological wellbeing in women with IBS and healthy women.
Peter, J., 2018 [62]	IBS *n* = 74	Cross-sectional and prospective longitudinal study	10-item CD-RISC	Principal component analysis, *t* Test, Mann–Whitney U test, chi-squared test	Resilience factors proved to be unidimensional in the total sample. Greater resilience and quality of life, and lower symptom severity and psychological distress were found after treatment.	To validate the construct and develop an integrational measure of various resilience domains by dimensional reduction, and to investigate changes in resilience in IBS patients after gut-directed hypnotherapy.
Dąbek-Drobny, 2020 [63]	IBS *n* = 129	Cross-sectional study	25-item RCS	Descriptive statistics, x2 test, Mann–Whitney test	A significant effect of resilience on IBS symptoms.	To assess the effect of selected personality traits and stress with IBS symptoms.
Parker, C. H., 2020 [64]	IBS *n* = 820 GP *n* = 1026	Online study survey—cross-sectional	CD-RISC	Descriptive statistics, ×2 or Fisher test, *t* Test or Wilcoxon, multivariable logistic/linear regression	Resilience was lower in IBS than the general population; and associated with worse IBS symptom severity. Global mental health affected resilience differently in IBS compared to the general population. Resilience scores were similar in IBS and other chronic GI conditions.	To compare resilience between IBS versus the general population and other chronic gastrointestinal conditions.

FSS—Functional Somatic Syndromes; IBS—Irritable Bowel Syndrome; CD-RISC—Connor-Davidson Resilience Scale; BRS—Brief Resilience Scale; GI—gastrointestinal; MANOVA—multivariate analysis of variance; IBD—Inflammatory Bowel Disorder, GP—general population, PCS—Pain Catastrophizing Scale; HCs—healthy controls; LGHS—Lang and Goulet Hardiness Scale.

## Data Availability

Not applicable.
